# Metabolomic Signature of Amino Acids, Biogenic Amines and Lipids in Blood Serum of Patients with Severe Osteoarthritis

**DOI:** 10.3390/metabo10080323

**Published:** 2020-08-08

**Authors:** Kaspar Tootsi, Kadri Vilba, Aare Märtson, Jaak Kals, Kaido Paapstel, Mihkel Zilmer

**Affiliations:** 1 Department of Traumatology and Orthopaedics, University of Tartu, 50090 Tartu, Estonia; kadri.vilba@kliinikum.ee (K.V.); aare.martson@kliinikum.ee (A.M.); 2 Endothelial Centre, University of Tartu, 50090 Tartu, Estonia; Jaak.kals@kliinikum.ee (J.K.); kaido.paapstel@kliinikum.ee (K.P.); mihkel.zilmer@ut.ee (M.Z.); 3 Traumatology and Orthopaedics Clinic, Tartu University Hospital, 50090 Tartu, Estonia; 4 Institute of Biomedicine and Translational Medicine, Department of Biochemistry, Centre of Excellence for Genomics and Translational Medicine, University of Tartu, 50090 Tartu, Estonia; 5 Department of Surgery, University of Tartu, 50090 Tartu, Estonia

**Keywords:** osteoarthritis, metabolites, metabolomics, lipidomics, amino acids, lipids, arginine, glycine, phosphatidylcholine, lysophosphatidylcholine

## Abstract

Metabolomic analysis is an emerging new diagnostic tool, which holds great potential for improving the understanding of osteoarthritis (OA)-caused metabolomic shifts associated with systemic inflammation and oxidative stress. The main aim of the study was to map the changes of amino acid, biogenic amine and complex lipid profiles in severe OA, where the shifts should be more eminent compared with early stages. The fasting serum of 70 knee and hip OA patients and 82 controls was assessed via a targeted approach using the AbsoluteIDQ™ p180 kit. Changes in the serum levels of amino acids, sphingomyelins, phoshatidylcholines and lysophosphatidylcholines of the OA patients compared with controls suggest systemic inflammation in severe OA patients. Furthermore, the decreased spermine to spermidine ratio indicates excessive oxidative stress to be associated with OA. Serum arginine level was positively correlated with radiographic severity of OA, potentially linking inflammation through NO synthesis to OA. Further, the level of glycine was negatively associated with the severity of OA, which might refer to glycine deficiency in severe OA. The current study demonstrates significant changes in the amino acid, biogenic amine and low-molecular weight lipid profiles of severe OA and provides new insights into the complex interplay between chronic inflammation, oxidative stress and OA.

## 1. Introduction

Osteoarthritis (OA) is considered the most prevalent joint disease and among the leading causes of disability in the elderly population. OA affects all tissues in and around the joint over time and leads to the development of distinctive clinical phenotypes depending on the most injured tissue. Even though OA has previously been considered as non-inflammatory local “wear and tear” damage of the joint, the understanding has changed and the role of systemic processes of the disease (low-grade inflammation, elevated oxidative stress) has been recognized. Although the pathomechanisms of OA are gaining much attention and there is progress in unraveling the mechanisms behind the disease, we are still currently lacking a disease-modifying therapy. One of the possible tools that could further our understanding of the disease is metabolomics.

Metabolomics is a new and emerging approach that investigates a considerable number of small metabolites that are the intermediates and end-products of many cellular processes. The metabolomic profile is the terminal downstream product of the genome. Investigation of the metabolome provides in-depth knowledge about the complex metabolic pathways and enables to gain profound insight into the pathogenesis of OA. 

Excessive oxidative stress (OxS) and systemic low-grade inflammation are found to be upregulated in OA [1]. Amino acids, polyamines and lipid compounds are closely involved in maintaining the balance between reactive oxygen species (ROS) and antioxidant systems. For example, spermine and spermidine act as free radical scavengers and their catabolism can be the source of toxic ROS [2]. Amino acids are also irreplaceable as building blocks of proteins and cell metabolism [3]. In recent years, a new concept has emerged that highlights functional amino acids, which participate in the regulation of key metabolic pathways to improve and maintain health, survival, growth, development and reproduction [4]. Several amino acids and their ratios have been suggested as biomarkers for OA [5,6]. Zhai et al. [6] have described in a two-stage study that the ratio of branched-chain amino acids to histidine (His) might serve as a biomarker for knee OA. Another study has found significantly lower levels of arginine (Arg) in knee OA patients and presented Arg as the most sensitive and specific biomarker discriminating OA from the healthy subjects [7]. These studies are a good example of the great potential of metabolomic studies.

Low-molecular weight bioactive lipid compounds have been shown to play a crucial role in regulating the immune response and systemic inflammation [8]. We have previously shown decreased levels of medium- and long-chain acylcarnitines in severe OA patients [9]. Several studies have found that low-molecular weight lipid levels and ratios are altered in OA patients [3,4,5,6]. Zhang et al. [10] found an increased ratio of lysophosphatidylcholine (lysoPC) to phosphatidylcholine (PC) in knee OA patients. Pousinis et al. [11] found that sphingolipids and PC were associated with OA in a mouse model. PCs are a major component of phospholipids that have an important role in joint lubrication [12]. The lysoPCs are biologically active lipids that have an important role in the regulation of inflammation and can induce cell death [13]. Nevertheless, further studies are needed to validate these new biomarkers and be able to implicate them in clinical decision-making.

The main aim of the study was to map the systemic changes of amino acids, biogenic amines and complex lipid profiles in end-stage OA compared with controls and identify potential systemic serum biomarkers that could be used in clinical practice.

## 2. Results

The study analyzed 70 end-stage OA patients and 82 age- and gender-matched controls. The general parameters of the study groups are presented in Table 1. There were no significant differences in age, gender proportions, total cholesterol, low-density lipoprotein (LDL) cholesterol and fasting glucose levels between the study groups. The OA group had a higher body mass index (BMI), high sensitive C-reactive protein (hs-CRP), white blood cell count (WBC) and triglycerides levels and lower high-density lipoprotein (HDL) cholesterol levels compared with the control group. 

Table 2 and Figure S1 present the comparison of amino acid levels between the OA and control group. There were significantly higher levels of Arg, asparagine (Asn), leucine (Leu), serine (Ser), asymmetric dimethylarginine (ADMA), phenylalanine (Phe) and spermidine, and lower levels of serotonin and spermine/spermidine ratio in the OA group after adjusting for BMI (Table 2). Since Arg is an important intermediate metabolite of the urea cycle, the comparison for Arg was adjusted for BMI and urea level that was significantly higher in the OA patients.

The complex lipid levels of both study groups are presented in Table 3 (significantly different) and Table S1 (with no significant differences). The lipid metabolites with a significant difference between the study groups are presented in Figure S2. The OA group had significantly lower serum levels of lysoPC a C14:0, PC aa C30:0, PC aa C32:2, PC aa C32:3, PC aa C34:3, PC aa C34:4, PC ae C30:0, PC ae C34:2 and PC ae C34:3, and higher levels of lysoPC a C20:4, PC aa C38:6, PC aa C40:6 and SM C20:2 compared with the control group (Figure S2).

**Figure 1:**
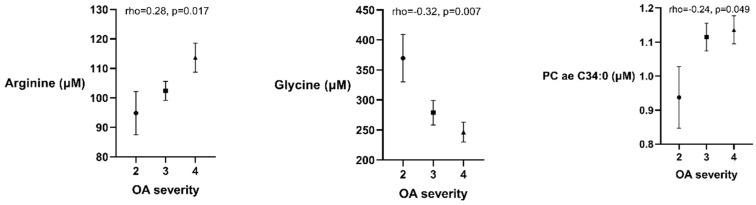
Plots (mean and standard error of the mean) describing the associations between osteoarthritis (OA) radiographic severity and arginine, glycine and phosphatidylcholine with 34 carbon molecules (PC ae 34:0) serum levels (μM). Concentrations of all metabolites are presented as µM.

Several metabolites were associated with the radiographic severity of OA in the univariate analysis: Arg (rho = 0.28, *p* = 0.017) and PC ae C34:0 (rho = 0.24, *p* = 0.049) had positive correlations, while Gly (rho = −0.32, *p* = 0.007) was negatively correlated with OA severity (Figure 1). These associations were further investigated in a stepwise multiple regression analysis. Gly and Arg were independently associated with OA severity (Table 4), however the association between PC ae C34:0 and OA radiographic severity was lost after including age, BMI, total cholesterol level and gender in the regression model. An increase of 1 grade in OA severity was equal to an average of a 10.0 μM higher level of serum Arg and 46.4 μM lower level of Gly while accounting for BMI, age and gender.

## 3. Discussion

OA is a complex disease and due to the lack of knowledge about the exact pathological mechanisms, there is currently no disease-modifying therapy. Advances in technology and research have led to new and promising methodologies. The multiomics approach enables to integrate big data from different fields of investigation (genomics, proteomics, transcriptomics, metabolomics) and further our understanding of the pathological mechanisms of different diseases. Investigating the systemic inflammation and excessive OxS related to OA using a personalized and multiomics approach is the most promising and innovative strategy [14,15]. The current study focuses on the targeted metabolomic assessment of severe OA patients. The present case–control study revealed alterations in serum amino acid and complex lipid profiles in the severe hip and knee OA patients compared with the control group. Furthermore, we demonstrate for the first time an independent association of Arg and Gly serum levels with OA radiographic severity (Table 4).

### 3.1. Amino Acids and Biogenic Amines

Amino acids have many irreplaceable functions in maintaining homeostasis: constituted as building blocks for proteins and enzymes, cell signaling, growth and reproduction [4]. Only a few studies have been conducted to assess the shifts in amino acid signatures in OA patients. We demonstrated significantly increased levels of Arg, Asn, Leu, Ser and Phe that helps to explain the systemic inflammatory state in OA. The level of polyamine spermidine was increased whereas the ratio of spermine to spermidine was decreased in the OA patients (Table 2), indicating a possible source of excessive OxS. 

Free **Arg** is acquired from diet, de novo endogenous synthesis (intestinal-renal axis) and protein turnover [16]. The significantly increased level of Arg in OA patients’ serum might be associated with the nitric oxide synthase (NOS) function. NOS releases nitric oxide (NO) from Arg and has an endogenous metabolic inhibitor ADMA. We also established an elevated level of ADMA in OA patients that suppresses NOS activity and results in declined utilization of Arg. However, Arg is also utilized by the synthesis of agmatine, creatine, urea and ornithine in the urea cycle which might impact the level of Arg in OA patients since they also had increased serum urea levels. Furthermore, we found that higher levels of Arg were associated with more severe radiographic OA (Figure 1). The role of NO in OA is controversial. NO is known to be associated with inflammation, however recent studies have highlighted the potential protective and anabolic properties [17,18,19]. In contrast to our results, some studies have found significantly lower levels of Arg in OA patients [7,20]. However, one of the mentioned studies by Pascale et al. [21] found elevated levels of Arg and ADMA in the synovial fluid which support the inhibitory effect of ADMA to NOS leading to Arg accumulation. Further, Chen et al. described significantly increased Arg serum levels in younger patients [20]. The discordance of Arg serum levels might arise from several reasons including different dietary intakes (Estonian vs. Canadian/Italian diet), pre-analytic protocols (storing samples in −70 °C vs. −20 °C), exclusion criteria and anthropometric parameters of study participants. Further, our study includes hip OA patients that had substantially higher Arg levels compared with knee OA patients (data not shown).

In addition, we found elevated **Asn** levels in OA patients in the current study. This might be associated with inflammation and protein breakdown in OA. The activity of Asn synthetase is associated with NO production and activity of macrophages [22,23]. Therefore, the higher levels of Asn might promote inflammation in OA patients. Interestingly, chondrocytes synthesize and secrete a cartilage-specific proteoglycan which have several types of carbohydrate chains attached to it, including Asn-linked oligosaccharides. Destructive processes of inflammation in OA may intensify the release of Asn from these complexes.

We found signs that the **Gly-Ser pathway** is impaired in OA patients. Gly is essential for type 2 collagen (prevalent in cartilage) synthesis in large quantities. Gly is mostly produced by enzyme Ser hydroxymethyltranferase (SHMT) that uses Ser as a substrate [24]. About 3 g of Gly per day is produced via biosynthesis that is only a small portion of a healthy person’s daily requirement (10 g) [24]. However, in OA the daily requirement may be substantially higher due to inflammatory and catabolic processes. We found that OA patients had higher levels of Ser and severe OA was associated with lower levels of Gly (Figure 1, Table 4). These changes suggest impaired SHMT function that leads to Gly deficiency and accumulation of Ser. In fact, the SHMT activity has been observed to be altered in obesity and related metabolic disorders that play an important role in OA development [25,26]. Our results are supported by a recent publication that found severe Gly deficiency in OA in an in vitro study [27]. Thus, increasing Gly dietary intake might promote collagen synthesis and the regeneration process in OA. 

In accordance with Zhai et al., the present study demonstrates increased **Leucine (Leu)** levels in OA patients [6]. Leu is an essential amino acid that is highly prevalent in muscle tissue and an important component in protein synthesis. Increased Leu in OA patients might indicate a higher rate of collagen breakdown. Some authors have suggested that the elevated Leu levels might be strongly associated with anterior cruciate ligament injury [28,29]. In contrast, our results with even higher levels of Leu in the hip OA patients (data not shown) suggest there are other mechanisms involved. In contrast to Zhai et al., we did not find a correlation between Leu, isoleucin or xleucin (combined leucine and isoleucine) with OA severity (data not shown) [6]. Possible causes of higher Leu levels in OA include increased collagen breakdown due to muscle atrophy and cartilage degradation. Partial oxidation of Leu has been shown to increase the release of ketone bodies (acetoacetate and 3-hydroxybutyrate) which are elevated in the urine of OA subjects [30]. However, specific pathways of higher Leu levels in OA patients remain to be elucidated.

The increased **spermidine** and lower **spermin to spermidine ratio** found in our study might indicate excessive OxS in OA. The spermine–spermidine system protects against OxS by scavenging free radicals and regulating other antioxidative mechanisms [31,32,33]. The increased level of spermidine in OA patients might be caused by the lower activity of spermine synthase, an enzyme that converts spermidine to spermine. Genetic mutation of spermine synthase leads to cytotoxic accumulation of spermidine in the cell and is characterized as Snyder–Robinson syndrome [32]. The accumulation of spermidine impairs lysosome function and leads to increased OxS [32]. We have recently demonstrated increased OxS and decreased total antioxidative capacity in OA patients which support the findings of the present study [1]. Thus, we link a new potential source of excessive OxS to OA.

Furthermore, the current study demonstrates a lower level of serum **serotonin** in OA patients (Table 2). Serotonin is an important regulatory monoamine. Tryptophan (Trp) is the main source of serotonin via hydroxylation and decarboxylation. Serotonin is mostly known for its function as a neurotransmitter, however it has been found to be involved in pain and inflammation in some types of arthritis [34]. Unfortunately, the role of serotonin in OA is largely unknown. In recent years, serotonin has been recognized as an important regulator of bone metabolism [35]. Lower levels of serotonin in OA might be associated with subchondral sclerosis and increased bone turnover but further studies are needed to clarify the specific mechanisms.

### 3.2. Lipidomic Analysis

Several studies have recognized the potential role of **glycerophospholipids** (including **PCs** and **lysoPCs**) and **sphingolipids** in the synovial fluid and less in the serum of OA subjects [5,11,12,36,37]. In support, we demonstrate altered levels of several PCs, lysoPCs and SMs in end-stage knee and hip OA patients (Table 3). Glycerophospholipids form the lipid bilayer and participate in cell signaling and membrane traffic regulation [38]. More specifically, PCs and SMs constitute over 50% of the cell membrane [39]. Phospholipids are metabolized by phospholipase A_1_ and A_2_ that hydrolyze the ester bonds of fatty acid chains linked to the glycerol backbone. The removal of the fatty acid chain results in lysoPCs formation. Phospholipids are one of the three major components of synovial fluid that act as a lubricant and also transport oxygen and nutrients to the cartilage [40]. Dysregulation of lipid metabolism has been proposed to be present in OA joints and represent an important pathophysiological feature of the disease [11,41,42]. LysoPCs are important components of oxidized LDL-cholesterol (oxLDL) that has been found to correlate with OA severity [1]. Thus, changes in several lysoPCs serum levels in OA patients might be associated with OxS. OxLDL is also an important activator of the inflammatory response that has a major role in the development and progression of OA [43]. Nevertheless, the results of studies investigating PCs and lysoPCs in OA are not uniform. We detected no significant difference in the lysoPCs to PCs ratio between the study groups (Table 3), while Zhang, et al. found the ratio to predict advanced knee OA and the future total knee replacement rate in these patients [10]. Furthermore, this ratio has been demonstrated to identify the responders to analgesic treatment [44]. Therefore, the role of PCs and lysoPCs in OA needs further research to untwine the different metabolic changes in OA subtypes. 

Sphingolipids (SM and SM (OH)) have a sphingoid base that is an organic aliphatic amino alcohol sphingosine or structurally similar compound [45]. These lipids are involved in several cellular processes that include proliferation, differentiation, apoptosis, stress response and cell senescence [36]. We found changes in OA patients’ serum levels of SM and SM (OH) that support their involvement in the pathogenesis of OA. Nevertheless, the specific functions of sphingolipids are yet to be discovered in OA. 

Limitations of the study include the inability to confirm cause and effect. There was no radiographic confirmation of the absence of OA in the control group which might introduce some false-negative OA subjects in the control group. The present study is cross-sectional and mostly serves as an exploratory study, therefore the results should be confirmed in longitudinal projects. Absence of a relevant control group has been a noteworthy problem in several previous metabolomic studies. The study groups in the present study were matched for age and gender but had a different BMI that might affect the levels of the metabolites even though it was accounted for in the statistical analysis. Although assessment of synovial fluid enables to assess the focal changes of the affected joint, synovial fluid aspiration is invasive and conveys various hazards and costs, thus would be hard to be implemented into a routine clinical practice as a diagnostic procedure of OA. Therefore, the present study focused on the serum of OA patients and proposes several low-molecular weight amino acids and complex lipids that help to identify OA from a healthy state.

In conclusion, the present study demonstrates altered levels of essential and functional amino acids, biogenic amines, several PCs and lysoPCs which indicate inflammation and excessive OxS in end-stage OA. We propose several novel metabolites that could serve as potential biomarkers.

## 4. Materials and Methods

### 4.1. Study Participants

End-stage knee and hip OA patients who met the American College of Rheumatology criteria for knee and hip OA were included in the study [46,47]. The patients were recruited prospectively from the Department of Orthopaedics, Tartu University Hospital. The exclusion criteria for the study were posttraumatic OA, infectious arthropathy, endocrine arthropathy, malignancy, acute inflammatory disease, insufficiency of kidneys (eGFR < 60 mL/min/1.73 m^2^), clinically significant heart failure and diabetes.

The control group was recruited from the same geographic region from the local family physicians. The controls were age- and gender-matched using a group matching strategy. The controls were chosen on the basis of an interview and clinical examination. The exclusion criteria for the control group were persistent knee or hip joint pain lasting over 1 month, acute or chronic inflammatory disease, diabetes, malignancy and renal insufficiency. 

### 4.2. Study Protocol

Lifestyle factors and medical history were obtained from the national and hospital electronic medical databases and from a patient-completed questionnaire. The blood samples were collected between 07:00 and 11:00 after an overnight fast and avoidance of alcohol and tobacco. Patients’ height and weight were recorded in the hospital. 

The study has been approved by the Ethics Committee on Human Research of the University of Tartu (approval number 230/T-2) and written informed consent was obtained from all study participants. 

### 4.3. Biochemical Analysis

Serum separator tubes (BD SST™ II Advance, Loughborough, UK) and plain tubes (Plain BD Vacutainer^®^ Tubes, Loughborough, UK) were used to draw blood for the clinical biochemistry analysis and for the detection of metabolites, respectively. All samples were centrifuged (3000 rpm for 15 min) at room temperature and the supernatant was collected into Eppendorff tubes and held at −70 °C until assessment. The concentrations of triglycerides, total cholesterol, LDL cholesterol, HDL cholesterol, glucose, white blood cell count and high-sensitivity C-reactive protein (hs-CRP) were measured in a local clinical laboratory with automated analyzers using standard laboratory methods. 

### 4.4. Targeted Metabolite Assessment

The levels of metabolites in the serum were determined using the AbsoluteIDQ™ p180 kit (BIOCRATES Life Sciences AG, Innsbruck, Austria) according to the manufacturer’s instructions. This assay allows the identification and quantification of up to 186 endogenous metabolites including 21 amino acids, 19 biogenic amines, 76 phosphatidylcholines, 14 lysophosphatidylcholines, 15 sphingomyelins, the sum of hexoses and 40 acylcarnitines. Glycerophospholipids are discriminated by the presence of ether (e) and ester (a) bonds in the glycerol moiety. Double letters (ae = acyl–alkyl, aa = diacyl) indicate that two glycerol positions are bound to a fatty acid chain, while a single letter (e = alkyl or a = acyl) indicates a bond with only one fatty acid chain. Identification and quantification of the metabolites was done using multiple reaction monitoring according to internal standards. The determination of the concentration of metabolites was done using the MetIDQ™ software package, which is an integral part of the AbsoluteIDQ kit. The concentrations of metabolites were calculated in μM. 

### 4.5. Radiological Measurement of Osteoarthritis Severity

Standard position preoperative weight-bearing X-rays were taken from the affected joints of the OA patients. The severity of OA was evaluated using the Kellgren–Lawrence scoring system [48] and evaluated separately by two raters who were blinded to the medical information. Consensus score was agreed by the raters and used in the analysis.

### 4.6. Statistical Analysis

Descriptive variables were presented as mean ± standard deviation for continuous and as percentages for categorical variables. Logarithmic transformation was used on data with non-Gaussian distribution. The metabolite serum concentrations were logarithmically transformed due to non-normal distribution. Student’s t-test and the Mann–Whitney U test were used for comparing the means of groups. The proportions between the study groups were evaluated with the Fisher’s exact or chi-square test. To account for the different BMI levels between the comparison groups, analysis of covariance was performed. The Spearman rank correlation was used to find potential associations between the severity of OA and the metabolites. Multivariate analysis was used with a stepwise) multiple linear regression to evaluate the independent predictors of radiographic severity of OA. Statistical analysis was performed using the SPSS software for Windows, version 22.0 (SPSS, Chicago, IL, USA).

## Figures and Tables

**Table 1:** General parameters.

Variable	Osteoarthritis (*n* = 70)	Controls (*n* = 82)	*p*-Value
Age (years)	62 ± 7	61 ± 8	0.173
Male/Female (*n*)	36/34	38/44	0.871
BMI (kg/m^2^)	27.8 ± 3.2	26.0 ± 3.5	0.001
hs-CRP (mg/L)	1.9 ± 1.1	1.5 ± 1.2	0.014
WBC (10^9^/L)	6.5 ± 1.4	5.7 ± 1.9	0.001
LDL-cholesterol (mmol/L)	4.0 ± 0.9	3.9 ± 1.1	0.804
Triglycerides (mmol/L)	1.7 ± 0.8	1.4 ± 1.1	0.001
HDL-cholesterol (mmol/L)	1.5 ± 0.5	1.7 ± 0.5	0.001
Total cholesterol (mmol/L)	5.8 ± 1.1	5.7 ± 1.3	0.539
Fasting glucose (mmol/L)	5.7 ± 0.7	5.7 ± 0.5	0.509
Urea (mmol/L)	6.1 ± 2.1	5.2 ± 1.2	0.014

Abbreviations: BMI—body mass index, hs-CRP—high-sensitivity C-reactive protein, WBC—white blood cell count, LDL—low-density lipoprotein, HDL—high-density lipoprotein.

**Table 2:** Amino acid and biogenic amine levels of osteoarthritis and the control group.

Variable	Osteoarthritis (*n* = 70)	Controls (*n* = 82)	*p*-Value
Alanine	430.9 ± 95.9	403.8 ± 89.5	0.172
Arginine	105.2 ± 22.4	96.3 ± 23.5	0.020 *
Asparagine	48.4 ± 8.3	44.1 ± 9.7	<0.001
Aspartate	28.0 ± 8.8	29.2 ± 10.8	0.692
Citrulline	36.8 ± 10.1	36.2 ± 9.7	0.302
Glutamine	703.9 ± 128.4	679.8 ± 131.5	0.130
Glutamate	50.7 ± 21.0	48.4 ± 20.6	0.872
Glycine	280.8 ± 116.2	289.8 ± 114.6	0.794
Histidine	106.3 ± 16.7	101.8 ± 16.1	0.185
Isoleucine	86.9 ± 25.5	79.9 ± 21.7	0.207
Leucine	180.9 ± 50.0	156.7 ± 39.5	0.008
Lysine	233.9 ± 42.2	224.4 ± 44.8	0.479
Methionine	24.3 ± 6.1	22.7 ± 5.7	0.137
Ornithine	92.8 ± 20.4	92.1 ± 23.2	0.892
Phenylalanine	74.1 ± 12.9	68.3 ± 11.8	0.016
Proline	162.3 ± 32.9	167.3 ± 48.2	0.352
Serine	137.4 ± 29.1	121.8 ± 27.6	0.001
Threonine	116.5 ± 21.8	118.0 ± 25.2	0.845
Tryptophan	68.6 ± 15.6	64.6 ± 14.7	0.220
Tyrosine	72.1 ± 16.8	67.3 ± 17.1	0.234
Valine	265.2 ± 60.3	254 ± 51.5	0.694
ADMA	0.618 ± 0.156	0.559 ± 0.133	0.037
Serotonin	0.576 ± 0.515	0.685 ± 0.280	0.004
Spermidine	0.188 ± 0.051	0.161 ± 0.046	0.001
Taurine	122.2 ± 32.0	124.2 ± 29.4	0.825
Spermine	0.158 ± 0.014	0.159 ± 0.016	0.677
Spermine-spermidine ratio	0.898 ± 0.227	1.060 ± 0.279	<0.001

Abbreviations: ADMA—asymmetric dimethylarginine. All variables are adjusted for body mass index. Concentrations of all metabolites are presented as µM ± standard deviation. *—adjusted for blood urea level and body mass index.

**Table 3:** Complex lipid profile of the osteoarthritis and the control group.

Metabolite	Control	Std	Osteoarthritis	Std	*p*-Value
lysoPC a C14:0	5.256	±0.873	4.748	±0.563	<0.001
lysoPC a C20:4	8.689	±2.414	9.843	±2.589	0.026
PC aa C30:0	4.143	±1.165	3.384	±0.77	<0.001
PC aa C32:2	3.069	±1.052	2.141	±0.794	<0.001
PC aa C32:3	0.424	±0.097	0.374	±0.085	0.010
PC aa C34:3	12.75	±3.573	10.64	±2.79	0.010
PC aa C34:4	1.367	±0.481	1.107	±0.379	<0.001
PC aa C38:6	76.57	±24.64	91.61	±25.77	0.010
PC aa C40:6	27.41	±9.457	33.12	±9.85	0.010
PC ae C30:0	0.430	±0.103	0.368	±0.075	<0.001
PC ae C34:2	9.942	±3.11	8.207	±1.757	0.010
PC ae C34:3	6.139	±2.02	4.768	±1.202	<0.001
SM C20:2	0.118	±0.051	0.143	±0.049	0.032

Group comparisons have been accounted for body mass index. Concentrations of all metabolites are presented as µM.

**Table 4:** Multiple regression analysis models for significant correlations from the univariate analysis.

(**a**) Regression Model with Glycine as the Dependent Variable in the Osteoarthritis Patients Group.				
**Variable**	**B**	**Std Error**	**Beta**	***p*-Value**
Constant	482.1	173.1		0.007
OA severity	−43.2	20.6	−0.25	0.040
Gender	−64.7	28.1	−0.28	0.025
Age	0.5	1.8	0.03	0.777
BMI	3.0	4.3	0.08	0.483
Glucose	−25–3	20.7	−0.15	0.227
(**b**) Regression Model with Arginine as the Dependent Variable in the Osteoarthritis Patients Group.				
**Variable**	**B**	**Std Error**	**Beta**	***p*-Value**
Constant	140.1	34.9		0.001
OA severity	9.12	4.1	0.28	0.031
Gender	5.77	5.6	0.13	0.310
Age	−0.31	0.37	−0.10	0.392
BMI	−0.24	0.86	−0.033	0.782
Glucose	−7.11	4.15	−0.21	0.092
(**c**) Regression Model with PC ae 34:0 as the Dependent Variable in the Osteoarthritis Patients Group.				
**Variable**	**B**	**Std Error**	**Beta**	***p*-Value**
Constant	0.440	0.34		0.196
OA severity	0.07	0.04	0.19	0.097
Gender	0.03	0.06	0.06	0.593
Age	−0.01	0.01	0.01	0.689
BMI	−0.01	0.01	−0.07	0.499
Cholesterol	0.115	0.026	0.50	<0.001

Abbreviations: OA—osteoarthritis, BMI—body mass index.

## Supplementary Materials

The following are available online at https://www.mdpi.com/2218-1989/10/8/323/s1, Table S1: Non-significantly different complex lipid profile of the osteoarthritis and the control group, Figure S1: Plots describing mean with standard error of the mean of amino acid, biogenic amine levels and spermine/spermidine ratio in the osteoarthritis and the control group, Figure S2: Plots describing mean with standard error of the mean of complex lipid metabolites (lysophosphatidylcholines (lysoPH), phosphatidylcholines (PC) and sphingomyelins (SM)) levels in the osteoarthritis and the control group.
