# Supplementary material for: Metabolomic Signature of Amino Acids, Biogenic Amines and Lipids in Blood Serum of Patients with Severe Osteoarthritis
Source: Metabolites. 2020 Aug 8;10(8):323. doi: 10.3390/metabo10080323 (PMC7464318; doi:10.3390/metabo10080323)
Supplement: Supplementary file 1 [file metabolites-10-00323-s001.pdf]

metabolites

MDPI

Table S1. Non significantly different complex lipid profile of the osteoarthritis and the control group

Metabolite	Control	Std	Osteoarthritis	Std	*p*-value
lysoPC a C16:0	152.4	± 35.7	160.3	± 26.3	0.093
lysoPC a C16:1	4.014	± 1.237	4.041	± 1.085	0.848
lysoPC a C17:0	2.924	± 0.891	2.868	± 0.739	0.774
lysoPC a C18:0	46.48	± 12.024	47.38	± 9.961	0.407
lysoPC a C18:1	32.90	± 9.212	30.99	± 6.903	0.907
lysoPC a C18:2	45.60	± 14.54	38.66	± 10.68	0.236
lysoPC a C20:3	2.950	± 0.921	3.151	± 0.824	0.189
lysoPC a C24:0	0.440	± 0.134	0.475	± 0.163	0.598
lysoPC a C26:0	0.766	± 0.314	0.839	± 0.331	0.626
lysoPC a C26:1	0.452	± 0.19	0.509	± 0.236	0.508
lysoPC a C28:0	0.493	± 0.161	0.496	± 0.155	0.907
lysoPC a C28:1	0.626	± 0.2	0.640	± 0.205	0.979
PC aa C24:0	0.376	± 0.154	0.421	± 0.172	0.563
PC aa C26:0	2.225	± 0.861	2.202	± 0.733	0.935
PC aa C28:1	3.431	± 0.733	3.422	± 0.794	0.783
PC aa C30:2	0.043	± 0.056	0.048	± 0.087	0.386
PC aa C32:0	10.97	± 2.369	10.76	± 2.077	0.907
PC aa C32:1	13.09	± 6.412	11.97	± 4.41	0.681
PC aa C34:1	178.3	± 48.03	177.4	± 36.96	0.848
PC aa C34:2	335.5	± 57.34	315.7	± 54.34	0.298
PC aa C36:0	2.288	± 0.668	2.312	± 0.585	0.745
PC aa C36:1	40.43	± 10.46	36.68	± 8.722	0.230
PC aa C36:2	218.1	± 42.677	201.4	± 42.25	0.184
PC aa C36:3	104.1	± 23.24	101.5	± 22.91	0.774
PC aa C36:4	139.4	± 36.52	151.8	± 36.64	0.298
PC aa C36:5	27.52	± 15.9	26.74	± 10.68	0.907
PC aa C36:6	0.980	± 0.411	0.841	± 0.288	0.184
PC aa C38:0	2.891	± 0.881	2.978	± 0.827	0.507
PC aa C38:1	1.115	± 0.387	1.069	± 0.372	0.984
PC aa C38:3	36.76	± 10.38	40.80	± 11.35	0.382
PC aa C38:4	79.88	± 23.091	92.73	± 26.05	0.084
PC aa C38:5	46.67	± 15.13	49.31	± 11.43	0.418
PC aa C40:1	0.409	± 0.074	0.414	± 0.08	0.774
PC aa C40:2	0.312	± 0.082	0.295	± 0.066	0.598
PC aa C40:3	0.492	± 0.147	0.485	± 0.094	0.828
PC aa C40:4	2.480	± 0.754	2.569	± 0.689	0.774
PC aa C40:5	7.327	± 2.094	7.865	± 2.297	0.558
PC aa C42:0	0.476	± 0.129	0.488	± 0.131	0.508
PC aa C42:1	0.234	± 0.061	0.245	± 0.057	0.233
PC aa C42:2	0.224	± 0.059	0.226	± 0.056	0.752
PC aa C42:4	0.141	± 0.031	0.140	± 0.032	0.907
PC aa C42:5	0.310	± 0.087	0.294	± 0.074	0.590
PC aa C42:6	0.570	± 0.158	0.545	± 0.107	0.774

PC ae C30:1	0.451	± 0.15	0.433	± 0.156	0.745
PC ae C30:2	0.132	± 0.034	0.138	± 0.036	0.876
PC ae C32:1	2.237	± 0.482	2.074	± 0.386	0.352
PC ae C32:2	0.646	± 0.152	0.607	± 0.113	0.558
PC ae C34:0	1.234	± 0.337	1.097	± 0.244	0.152
PC ae C34:1	8.030	± 1.854	7.350	± 1.406	0.321
PC ae C36:0	0.759	± 0.186	0.749	± 0.16	0.907
PC ae C36:1	6.640	± 1.595	6.185	± 1.369	0.460
PC ae C36:2	11.80	± 2.992	10.32	± 2.285	0.117
PC ae C36:3	6.542	± 1.885	5.595	± 1.14	0.075
PC ae C36:4	12.95	± 3.808	13.06	± 3.489	0.876
PC ae C36:5	9.151	± 2.634	8.975	± 2.106	0.987
PC ae C38:0	2.080	± 0.689	1.963	± 0.503	0.774
PC ae C38:1	0.293	± 0.181	0.288	± 0.176	0.907
PC ae C38:2	1.631	± 0.408	1.432	± 0.32	0.122
PC ae C38:3	3.184	± 0.755	3.123	± 0.639	0.907
PC ae C38:4	10.10	± 2.465	10.20	± 2.363	0.876
PC ae C38:5	14.49	± 3.866	14.57	± 3.194	0.774
PC ae C38:6	6.615	± 1.974	6.743	± 1.685	0.670
PC ae C40:1	1.358	± 0.304	1.291	± 0.28	0.681
PC ae C40:2	1.549	± 0.356	1.581	± 0.41	0.935
PC ae C40:3	0.779	± 0.15	0.762	± 0.133	0.886
PC ae C40:4	1.819	± 0.389	1.809	± 0.369	0.876
PC ae C40:5	2.836	± 0.634	2.826	± 0.563	0.875
PC ae C40:6	4.322	± 1.117	4.513	± 1.149	0.407
PC ae C42:0	0.862	± 0.101	0.902	± 0.096	0.093
PC ae C42:1	0.478	± 0.119	0.510	± 0.127	0.407
PC ae C42:2	0.547	± 0.119	0.542	± 0.107	0.982
PC ae C42:3	0.755	± 0.159	0.752	± 0.159	0.876
PC ae C42:4	0.690	± 0.151	0.655	± 0.148	0.828
PC ae C42:5	1.698	± 0.339	1.680	± 0.332	0.907
PC ae C44:3	0.135	± 0.039	0.138	± 0.038	0.710
PC ae C44:4	0.318	± 0.064	0.306	± 0.067	0.848
PC ae C44:5	1.411	± 0.35	1.397	± 0.34	0.875
PC ae C44:6	0.930	± 0.228	0.952	± 0.223	0.460
SM (OH) C14:1	3.425	± 0.828	3.273	± 0.89	0.558
SM (OH) C16:1	1.548	± 0.402	1.551	± 0.429	0.907
SM (OH) C22:1	6.251	± 1.327	5.807	± 1.265	0.155
SM (OH) C22:2	5.177	± 1.299	5.021	± 1.197	0.746
SM (OH) C24:1	0.612	± 0.146	0.570	± 0.12	0.243
SM C16:0	53.70	± 10.26	54.32	± 9.04	0.626
SM C16:1	7.694	± 1.798	8.098	± 1.485	0.407
SM C18:0	9.86	± 2.463	10.77	± 2.554	0.321
SM C18:1	4.675	± 1.305	5.261	± 1.342	0.181
SM C24:0	9.122	± 1.915	8.798	± 1.622	0.626
SM C24:1	24.65	± 5.51	25.81	± 5.169	0.372
SM C26:0	0.087	± 0.029	0.081	± 0.022	0.598
SM C26:1	0.213	± 0.053	0.225	± 0.059	0.460
Total lysoPC/PC	0.20	± 0.203	0.203	± 0.028	0.774

Group comparisons have been accounted for body mass index. Concentrations of all metabolites are presented as μM.

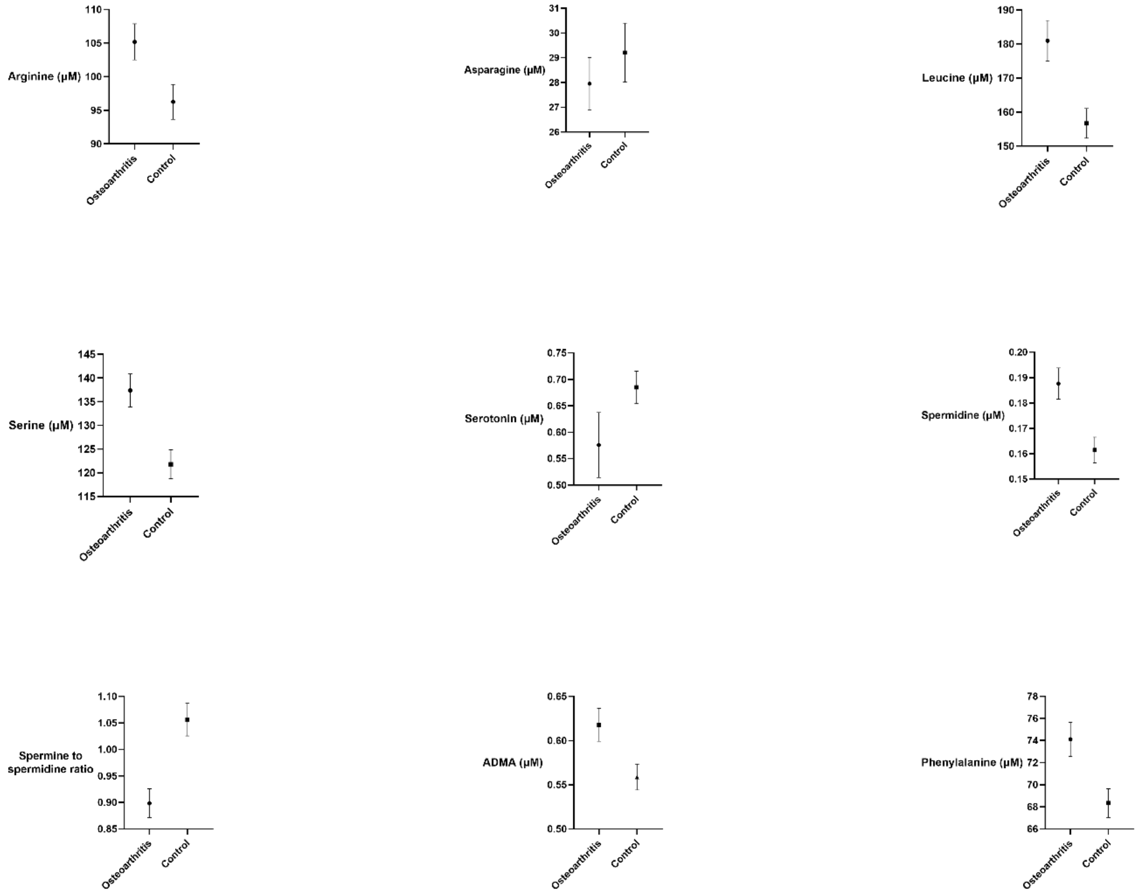

Figure S1. Plots describing mean with standard error of the mean of amino acid, biogenic amine levels and spermine/spermidine ratio in the osteoarthritis and the control group. The concentrations of metabolites are presented as μM.

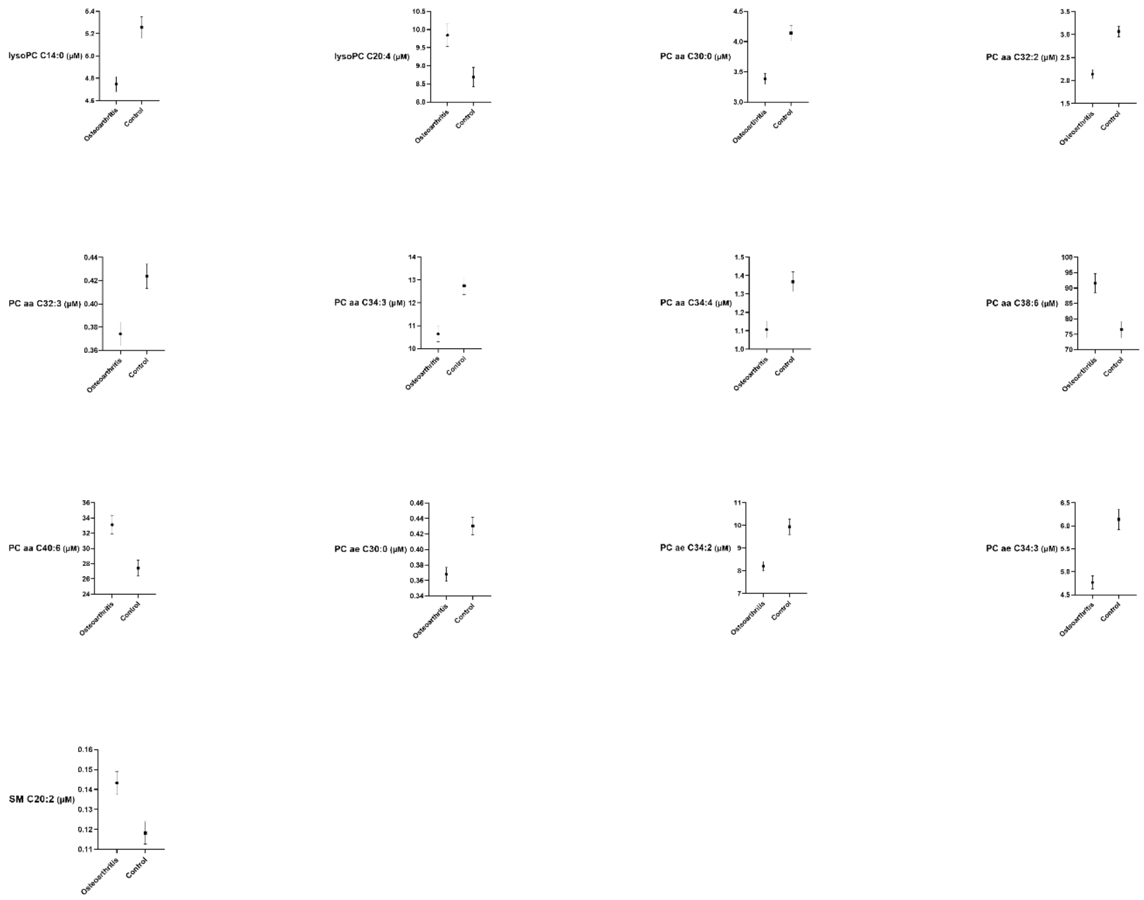

Figure S2. Plots describing mean with standard error of the mean of complex lipid metabolites (lysophosphatidylcholines (lysoPH), phosphatidylcholines (PC) and sphingomyelins (SM)) levels in the osteoarthritis and the control group. The concentrations of all metabolites are presented as μM.
